# Diagnosing milk aspiration as a cause of death in sudden unexpected infant death: forensic insights from post-mortem analysis impacting criminal investigations

**DOI:** 10.1007/s12024-025-00958-0

**Published:** 2025-01-31

**Authors:** Alessandro Mauro Tavone, Francesca Servadei, Francesca Cazzato, Erica Giacobbi, Rita Bonfiglio, Antonio Oliva, Gian Luca Marella

**Affiliations:** 1 Department of Surgical Sciences, University of Rome Tor Vergata, Via Montpellier 1, 00133 Rome, Italy; 2 Department of Experimental Medicine, Torvergata Oncoscience Research, University of Rome “Tor Vergata”, Via Montpellier 1, 00133 Rome, Italy; 3 Department of Health Surveillance and Bioethics, Section of Legal Medicine, Fondazione Policlinico A. Gemelli IRCCS, Università Cattolica del Sacro Cuore, Largo Francesco Vito 1, 00168 Rome, Italy

**Keywords:** Sudden unexpected infant death, Milk aspiration, Immunoschemistry, Forensic autopsy

## Abstract

Sudden unexpected infant death (SUID) encompasses both explained and unexplained infant fatalities. When a comprehensive investigation yields inconclusive results, the case is classified as sudden infant death syndrome (SIDS). On the other hand, the most frequent non-SIDS diagnoses may be attributed to specific causes of death including a heterogeneous spectrum of conditions and disorders (e.g., trauma, asphyxia, suffocation, infection and metabolic diseases). Although rare, milk aspiration is a recognized cause of SUID that can lead to acute respiratory failure. This case report describes the death of a three-month-old infant found unresponsive in a traditional African baby carrier. Gross examination revealed no significant anomalies other than increased lung weight and the presence of milk-like material in the airways, alveoli, and stomach. Histological and ultrastructural analyses identified granular brownish material with birefringent globules in the lungs, consistent with aspirated milk. Immunohistochemical staining was positive for beta-lactoglobulin, confirming formula milk aspiration. This evidence was crucial in excluding maternal negligence as a cause of death, instead supporting an ante-mortem aspiration event resulting from regurgitation. This case highlights the diagnostic challenges associated with fatal milk aspiration and emphasizes the critical importance of a multidisciplinary approach. The integration of clinical history, autopsy findings, and advanced histopathological techniques is essential for accurately determining the cause of death and ensuring a sound legal assessment within the Courtroom setting.

## Introduction

Sudden unexpected infant death (SUID) encompasses any unexpected fatal event in infancy, covering both explained and unexplained deaths. When a comprehensive forensic investigation, including a full autopsy with toxicological, histological, and microbiological analyses, along with a review of the clinical history, fails to determine the cause of death, it is classified as sudden infant death syndrome (SIDS) [1–3]. On the other hand, the most common non-SIDS diagnoses are typically associated with identifiable causes of death, encompassing a heterogeneous spectrum of conditions and disorders (e.g., trauma, asphyxia, suffocation, infection and metabolic diseases) [1, 4]. In such scenarios, components from the infant’s gastric content are often detected in the pulmonary airways during autopsy on infants who have died suddenly. This phenomenon, known as “*gastric aspiration*”, occurs when stomach contents are inhaled into the lungs, potentially leading to severe complications [5]. In infants under one year of age, milk represents the most frequently aspirated food substance [5]. Milk aspiration, although rare, is a potential cause of sudden unexpected infant death, that may occur when milk is inhaled into the airways, leading to acute respiratory failure and death. Such deaths are primarily associated with severe gastroesophageal reflux [6, 7], but may also result from improper positioning during sleep or feeding [8]. Additionally, milk aspiration can occur in individuals affected by swallowing disorders, such as those with Prader-Willi Syndrome [7] or other genetic disorders [9, 10]. Despite their significant impact on society, the incidence of fatal milk aspiration cases in infants is likely underestimated. Accurate post-mortem diagnosis is challenging for forensic pathologists and may require both non-routine investigations and a multidisciplinary assessment [6]. One of the primary diagnostic challenges lies in distinguishing between ante-mortem and post-mortem milk aspiration. Indeed, post-mortem aspiration can occur during cardiopulmonary resuscitation or as a result of terminal agonal reflexes [8, 11]. Moreover, establishing whether the aspiration is the cause of death or merely an incidental finding can be complex. This determination is typically achieved through the integration of forensic evidence and circumstantial information [9, 12]. Accurate responses to these diagnostic challenges are paramount, as erroneous forensic conclusions regarding the cause of death could result in incorrect legal determinations. We reported the case of a three-month-old female infant found dead by her mother inside a traditional African baby carrier. A comprehensive forensic autopsy and conventional post-mortem analyses were supplemented with additional diagnostic examinations (i.e., immunohistochemistry and electron microscopy), leading to the determination of the cause of the infant’s death. The aim of our study is to show how the application of specialized forensic techniques combined with a multidisciplinary approach, significantly impacted the forensic investigation and the subsequent legal determination in the Courtroom.

## Case presentation

### Case report

A three-month-old female infant was found deceased by her mother inside a traditional African baby carrier (also known as “*kitenge*”) after a 40-min bus journey along a predominantly well-paved and straight road. Despite faint cries heard during the bus journey, upon arrival at destination, the mother found the baby unresponsive with nasal secretions. Emergency services were alerted, and upon arrival, found the child in cardiopulmonary arrest. Despite resuscitation efforts, the death was pronounced shortly thereafter. The mother reported standing throughout the journey to avoid compressing her daughter, who was carried on her back, and informed law enforcement that the infant had not sustained any impact during transit. However, the woman was subsequently investigated by the Public Prosecutor’s Office on charges of manslaughter involving her daughter. The infant’s clinical history was unremarkable except for a three-day episode of persistent cough and inconsolable crying, which led to hospitalization on the 15th day after birth. Upon admission, bilateral fine crackles were noted, and oxygen saturation (SaO_2_) was recorded at 94%. The infant was discharged after six days with stabilized parameters and a clinical diagnosis of bronchiolitis. Before the forensic autopsy, post-mortem nasopharyngeal swabs were collected for microbiological analysis [13], which revealed rhinovirus/enterovirus positivity via real-time PCR.

### Autopsy findings

The autopsy revealed a female infant without dysmorphisms in the craniofacial complex, exhibiting below-average growth according to the World Health Organization (WHO) growth curves [14] (age: 91 days; weight: 4.85 kg – 7.2nd percentile; crown-heel length: 56 cm – 3.5th percentile).

At the external examination, a small amount of whitish material was observed leaking from the nasal orifices, along with bilateral subungual cyanosis of the hands.

The autopsy did not reveal any significant gross anomalies, except for an increase in the weight and consistency of the lungs (lungs weights: right-59.0 g; left-42.7 g; reference values: right-35.0 g; left-30.0 g [15]), bilateral subpleural petechiae, and multi-visceral congestion. Upon sectioning of the trachea, a whitish, milky substance was detected on the mucosal surface. Similar whitish material was also observed in the esophageal lumen and in considerable quantities in the stomach.

### Histological, immunohistochemical, and ultrastructural findings

**Fig. 1:**
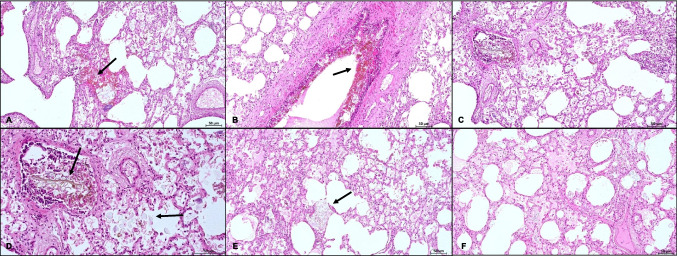
**A** Granular brownish material (arrow) mixed with hyaline translucent globules in the lumen of alveolar spaces of the left lung parenchyma, with bacterial aggregates and sparse macrophages (HE, original magnification 10x); **B** Brownish granular material (arrow) adherent to bronchial mucosa (HE, original magnification 10x); **C** Aggregates of brownish granular material mixed with hyaline globules in the bronchiolar lumen and alveolar spaces. Aggregates of macrophages and lymphocytes on the right side of the panel. An elongated structure with a reticular morphology compatible with a vegetable fiber in the bronchiolar lumen (HE, original magnification 10x); **D **Higher magnification of image **C**, highlighting the presence of hyaline translucent globules (right arrow) associated to brownish granular material ad an elongated structure compatible with a vegetable fiber (left arrow) (HE, original magnification 20x); **E **Granular brownish material mixed with hyaline translucent globules (arrow) in the lumen of alveolar spaces and macrophages with granular cytoplasm in another field of pulmonary parenchyma (HE, original magnification 10x); **F **Alveolar edema associated with granular brownish material and macrophages (HE, original magnification 10x)

The histological examination of the lungs (i.e., hematoxylin and eosin staining (HE)) revealed bilateral, ubiquitarian, weakly eosinophilic, often brownish granular, amorphous material, mixed with birefringent translucent globules in the lumens of bronchial and bronchiolar structures, and alveolar spaces accompanied by interalveolar histiocytes with granular cytoplasm, occasional small lymphocytic aggregates, and focal endoalveolar hemorrhages (Fig. 1A-F).

**Fig. 2:**
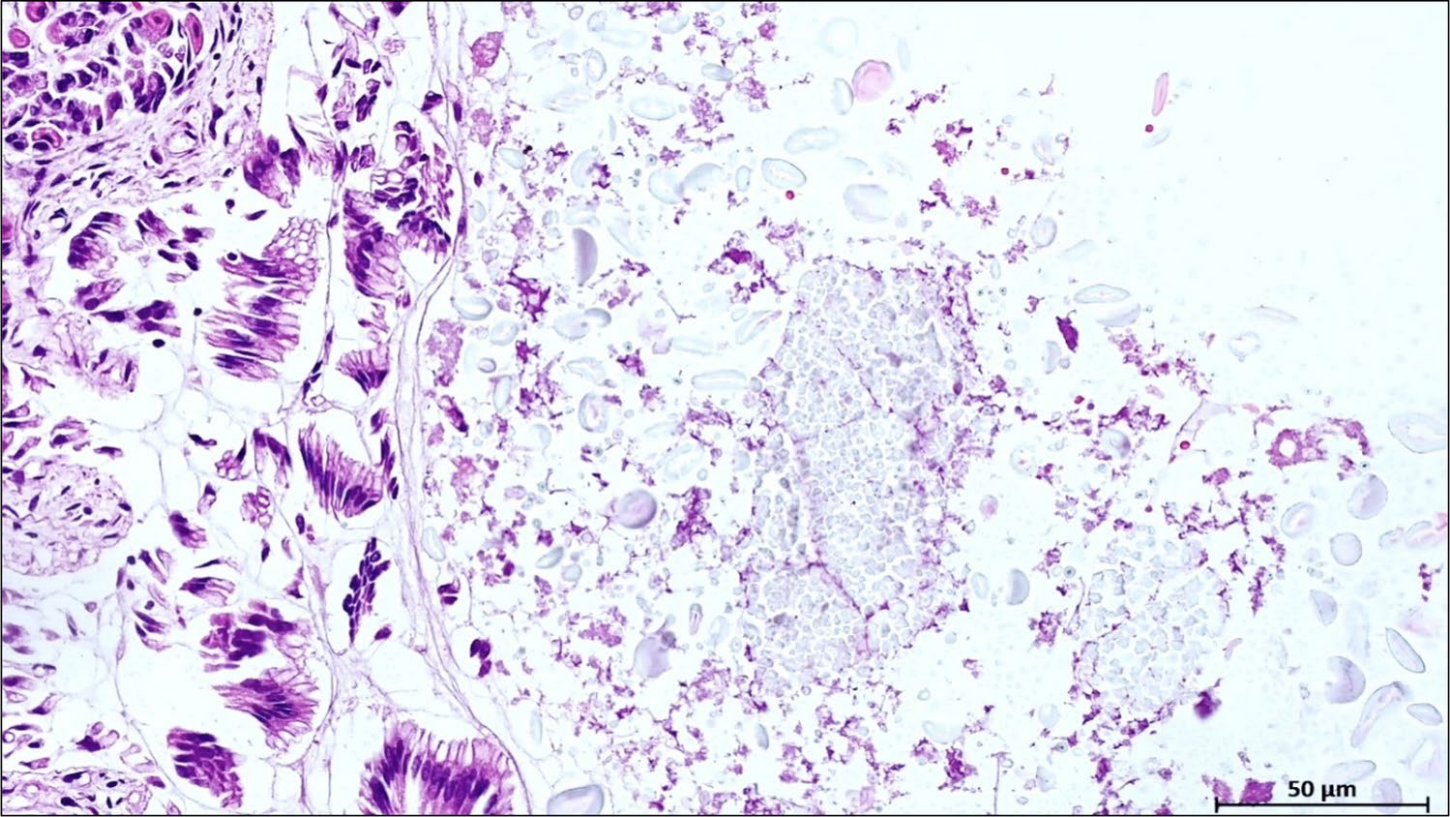
Granular material with hyaline translucent granules in the gastric lumen (HE, original magnification 10x)

Material with the same characteristics (i.e., amorphous material with birefringent globules) was found also in the stomach (Fig. 2).

**Fig. 3:**
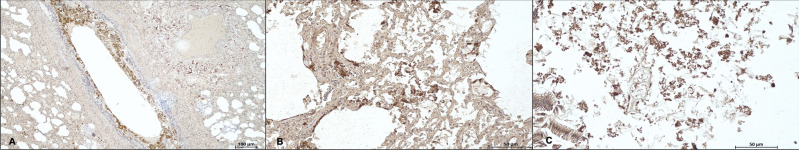
**A-B** Granular material in bronchial mucosa and alveolar spaces, highlighted in dark brown by beta-lactoglobulin antibody (Bethyl Laboratories Bovine Beta-Lactoglobulin Polyclonal Antibody A10-125A, original magnification 5 × in (**A**) and 10 × in (**B**)); **C** An elongated fibrillary structure consistent with a vegetable fiber in the stomach (Bethyl Laboratories Bovine Beta-Lactoglobulin Polyclonal Antibody A10-125A, original magnification 10x)

Immunohistochemical analysis using the Bethyl Laboratories Bovine Beta-Lactoglobulin Polyclonal Antibody A10-125A [16], revealed beta-lactoglobulin positivity in the gastric, and in both intrabronchial and intra-alveolar amorphous material (Fig. 3).

Anti-alpha-lactalbumin testing (Invitrogen LALBA Antibody PA5-76868) yielded inconclusive results due to nonspecific background staining.

**Fig. 4:**
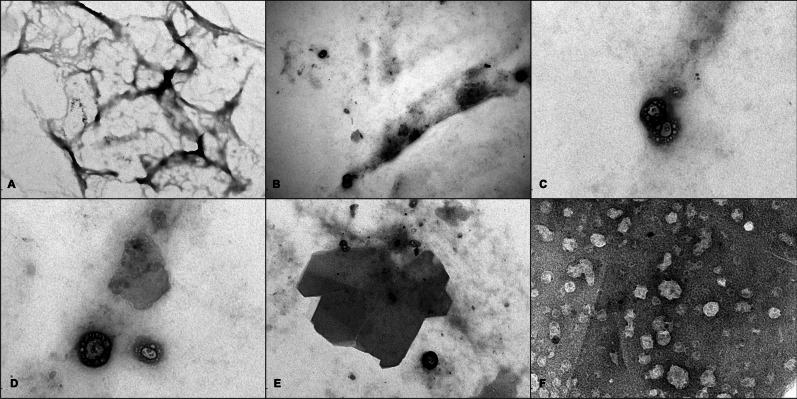
Ultrastructural examination of seriated lung parenchyma sections with the presence of presumably organic residues in pulmonary alveoli **(A-B)**, characterized by a reticular appearance, characteristic of various organic compounds, including carbohydrate polysaccharides like starch. Cellular debris **(C-D)** and crystals **(E–F)** near these structures

Ultrastructural examination performed using FEI 268D transmission electron microscope on the pulmonary tissue confirmed the presence of residues of presumably organic nature in the pulmonary alveoli (Fig. 4A-B). These structures displayed a reticular appearance, characteristic of various organic compounds, including polysaccharide carbohydrates such as starch [17].

Near these structures, cellular debris (Fig. 4C-D) and crystals (Fig. 4E–F) were frequently observed.

Histopathological examination of the other organs, using hematoxylin and eosin staining, revealed no significant pathological changes, except from moderate blood congestion observed in certain organs including the thymus, heart, liver, adrenal glands, and kidneys.

## Discussion

### Forensic evidence in fatal milk-aspiration

We reported the case of a three-month-old female infant found deceased after being transported in a traditional African baby carrier, with an otherwise unremarkable clinical history. At autopsy, no significant gross abnormalities were found, except for a slight increase in the weight and consistency of the lungs and the detection of whitish, milky material in the lumen of the trachea, esophageal lumen and stomach. Histological examination of the lungs (i.e., hematoxylin and eosin staining) revealed bilateral, widespread, weakly eosinophilic, often brownish granular, amorphous material mixed with birefringent translucent globules within bronchial and bronchiolar lumens, as well as alveolar spaces. This was associated with intralveolar histiocytes displaying granular cytoplasm, occasional small lymphocytic aggregates, and focal endoalveolar hemorrhages. Material with the same characteristics was also detected in the stomach. These histological findings, with evidence of the same material in both stomach and lungs, suggested milk aspiration, likely resulting from regurgitation of gastric contents, as the cause of death. The gross and microscopic findings align with previous reports [8, 9, 12, 18] describing milk aspiration as a cause of acute respiratory failure due to mechanical airway obstruction and potential chemical irritation from gastric contents.

### Post-mortem diagnostic challenges

Post-mortem diagnosis of fatal milk aspiration and its causal inference in determining the cause of death pose a significant challenge for the forensic pathologist. Indeed, conventional forensic examinations alone may yield negative or inconclusive results. At gross examination, fatal milk aspiration may show the presence of white milk-like liquid material in the nostrils, mouth, trachea, esophagus and stomach [8, 12]. Other non-specific findings may include hyperemia and oedema of the tracheal mucosa [12] and pleural petechiae [9]. The histopathological analysis often shows amorphous material, finely granular eosinophilic material or weakly basophilic particles in the alveoli and bronchioles [5, 8, 12]. This material may be associated with inflammatory cells [12], suggesting a response to the aspiration. However, the absence of significant inflammation might indicate a rapid or sudden death [8]. Clumps of bacteria may also be histologically detected [5]. The presence of lipid-laden alveolar macrophages may indicate the inhalation of lipid-containing substances, aligning with the presence of aspirated milk in suspected cases [9, 12]. In addition, distinguishing milk from proteinaceous edematous fluid, fibrin, and other airway debris becomes increasingly challenging over time due to bacterial and chemical modification [5]. In our case, the extensive distribution of the material throughout all lung fields classified the aspiration as severe [5]. Hence, additional post-mortem diagnostic tests were performed, supplementing standard histopathological analyses, to investigate the nature of the material found.

### Milk type detection through immunohistochemical and ultrastructural analysis

The mother’s refusal to disclose the type of milk used to feed the infant posed an additional diagnostic challenge, requiring the use of immunohistochemical markers able to identify both feeding options, i.e. human breast milk and formula milk. Immunohistochemical analysis represents a reliable diagnostic tool in post-mortem tissues [19] to detect fatal milk aspiration and distinguishing between formula milk and human breast milk. Beta-lactoglobulin, a major whey protein found in cow’s milk, serves as a specific marker for formula milk since it is absent in human breast milk [20, 21]. Immunostaining for beta-lactoglobulin produces a distinct reaction, making it a reliable indicator of formula milk aspiration [9]. Conversely, alpha-lactalbumin, present in both human and cow’s milk, reacts with both types of milk proteins, leading to cross-reactivity and potential nonspecific background staining, thus limiting its use for differentiating between the two types of milk. In our case, immunohistochemical analysis provided crucial information about the nature of the aspirated material, revealing positive results for beta-lactoglobulin and inconclusive results for alpha-lactalbumin. The positivity for beta-lactoglobulin in both gastric and pulmonary samples confirmed the ingestion and subsequent aspiration of formula milk. The detection of translucent hyaline globules and fibrillary fragments, presumably of vegetable origin, suggested possible contamination or additives in the formula, such as milk thickeners. This finding raises concerns about the quality and safety of the formula used. To test the hypothesis that the birefringent hyaline globules represent additives for infant formula, acting as milk thickeners such as starch, we performed an electron microscopy study on lung tissue. In support of our hypothesis, these globules exhibited a reticular appearance at the ultrastructural level, characteristic of various organic compounds, including carbohydrate polysaccharides such as starch.

### Distinguishing ante-mortem from post-mortem milk aspiration

The main diagnostic challenge was determining whether milk aspiration occurred ante-mortem or post-mortem. Microscopic detection of milk not only in the proximal airways but, most importantly, in the distal airspaces and across multiple examined lung fields, along with the presence of reactive macrophages, strongly suggested ante-mortem aspiration as the primary cause of death. A potential role of respiratory infection in the cause of death could not be excluded, as the post-mortem nasopharyngeal swab tested positive for rhinovirus/enterovirus. Notably, at 15 days of age, the infant was hospitalized for dyspnea and persistent cough. At that time, she was treated with oxygen and discharged with a clinical diagnosis of suspected bronchiolitis. It can be hypothesized that bronchiolitis may be related to an episode of gastric content aspiration secondary to gastroesophageal reflux. Furthermore, gastroesophageal reflux, central apnea, and laryngospasm are common in infants with respiratory infections, potentially exacerbating the risk of aspiration [22]. Focusing on the circumstances surrounding the death, it remains uncertain whether the bus experienced significant vibrations or shaking during the journey. However, it should be considered that physiological gastroesophageal reflux peaks around 2–3 months of age [23] due to the immaturity of the critical components of the anti-reflux barrier (e.g., lower esophageal sphincter and crural diaphragm) [24]. Thus, it can’t be ruled out that even minimal movements during the bus journey may have contributed to reflux-related complications, potentially acting as a predisposing factor for milk aspiration. Moreover, the use of the soft back carrier for infant transport raised additional concerns [15]. It is noteworthy that the US Consumer Product Safety Commission (CPSC) warns about suffocation risks for infants, especially those under 4 months, when using infant sling carriers [25, 26]. However, to the best of our knowledge, there is no robust scientific evidence directly linking soft carriers to asphyxiation in infants. Overall, our comprehensive forensic investigation, including additional non-routine examinations, allowed us to state that the infant’s death was caused by acute respiratory failure due to the aspiration of formula milk into the airways and alveolar spaces, following gastric content regurgitation. The death due to respiratory failure is supported by the extensive presence of material in lung samples, especially at the endoalveolar level. This evidence suggests that the infant was alive and breathing independently, rather than passive inhalation occurring. This evidence exonerated the mother from criminal liability, as the aspiration was determined to be an ante-mortem event caused by regurgitation rather than the result of improper feeding, external compression, or suffocation by the soft carrier or the adult’s body.

## Conclusions

Our case underscores the importance of a thorough and multidisciplinary approach in diagnosing milk aspiration as a cause of SUID. The integration of clinical history, autopsy findings, and advanced histopathological diagnostic techniques, such as immunohistochemistry and electron microscopy, is essential for differentiating between ante-mortem and post-mortem events, as well as for identifying the inhaled or ingested material. This approach not only provides clarity on the cause of death but also ensures that criminal liabilities are accurately determined in the Courtroom. Future research should focus on improving specificity of human breast milk markers for immunohistochemical analyses and understanding the role of environmental and physiological factors in infant deaths related to milk aspiration.

### Key points

1. Histological and ultrastructural analyses revealed milk aspiration as the cause of death.
2. Immunohistochemistry confirmed formula milk aspiration through beta-lactoglobulin detection.
3. Microscopic findings indicated ante-mortem aspiration due to gastric regurgitation.
4. Forensic evidence led to exoneration of the mother from criminal liability.

## Data Availability

The data will be made available upon reasonable request.

## References

[1] Moon RY, Task force on sudden infant death syndrome, Darnall RA, Feldman-Winter L, Goodstein MH, Hauck FR. SIDS and other sleep-related infant deaths: evidence base for 2016 updated recommendations for a safe infant sleeping environment. Pediatrics. 2016;138. 10.1542/peds.2016-2940. 10.1542/peds.2016-2940 27940805

[2] Krous HF, Beckwith JB, Byard RW, Rognum TO, Bajanowski T, Corey T, et al. Sudden Infant Death Syndrome and Unclassified Sudden Infant Deaths: A Definitional and Diagnostic Approach. Pediatrics. 2004;114:234–8. 15231934 10.1542/peds.114.1.234

[3] Cazzato F, Coll M, Grassi S, Fernàndez-Falgueras A, Nogué-Navarro L, Iglesias A, et al. Investigating cardiac genetic background in sudden infant death syndrome (SIDS). Int J Legal Med. 2024;138(6):2229–37. 38849547 10.1007/s00414-024-03264-6 PMC11490465

[4] Loughrey CM, Preece MA, Green A. Sudden unexpected death in infancy (SUDI). J Clin Pathol. 2005;58:20–1. 15623476 10.1136/jcp.2004.020677 PMC1770550

[5] Alex N, Thompson JM, Becroft DM, Mitchell EA. Pulmonary aspiration of gastric contents and the sudden infant death syndrome. J Paediatr Child Health. 2005;41:428–31. 16101977 10.1111/j.1440-1754.2005.00660.x

[6] Iwadate K, Doy M, Ito Y. Screening of milk aspiration in 105 infant death cases by immunostaining with anti-human α-lactalbumin antibody. Forensic Sci Int. 2001;122:95–100. 11672962 10.1016/s0379-0738(01)00469-8

[7] Leape LL, Holder TM, Franklin JD, Amoury RA, Ashcraft KW. Respiratory arrest in infants secondary to gastroesophageal reflux. Pediatrics. 1977;60:924–8. 600609

[8] Kibayashi K, Iwadate K, Shojo H. 4. Milk Aspiration in an Infant During Supine Bottle Feeding. Med Sci Law. 2004;44:272–5. 15296253 10.1258/rsmmsl.44.3.272

[9] Osawa M, Ikeda H, Ueda A, Naito H, Nagao R, Kakimoto Y. Gastric aspiration in sudden unexpected infant death of Prader-Willi syndrome: immunohistochemical detection of feeding components. Int J Legal Med. 2022;136:1883–8. 36018383 10.1007/s00414-022-02883-1 PMC9576639

[10] Castori M, Servadei F, Laino L, Pascolini G, Fabbri R, Cifani AE, et al. Axial skeletogenesis in human autosomal aneuploidies: A radiographic study of 145 second trimester fetuses. Am J Med Genet A. 2016;170:676–87. 26687031 10.1002/ajmg.a.37510

[11] Byard R, Beal S. Gastric aspiration and sleeping position in infancy and early childhood. J Paediatr Child Health. 2000;36:403–5. 10940183 10.1046/j.1440-1754.2000.00503.x

[12] Maiese A, La RR, Arcangeli M, Volonnino G, De MA, Frati P, et al. Multidisciplinary approach to suspected sudden unexpected infant death caused by milk-aspiration: A case report. World J Clin Cases. 2020;8:4128–34. 33024771 10.12998/wjcc.v8.i18.4128 PMC7520785

[13] Servadei F, Mauriello S, Scimeca M, Caggiano B, Ciotti M, Anemona L, et al. Persistence of SARS-CoV-2 Viral RNA in Nasopharyngeal Swabs after Death: An Observational Study. Microorganisms. 2021;9:800. 33920259 10.3390/microorganisms9040800 PMC8103507

[14] de Onis M, Garza C, Victora CG, Onyango AW, Frongillo EA, Martines J. The who multicentre growth reference study: planning, study design, and methodology. Food Nutr Bull. 2004;25(1 Suppl):S15–S26. 10.1177/15648265040251S103. 10.1177/15648265040251S103 15069916

[15] Gilbert-Barness E. Potter’s pathology of the fetus, infant and child. 2nd ed. Philadelphia: Mosby Elsevier; 2007.

[16] Scimeca M, Montanaro M, Bonfiglio R, Anemona L, Agrò EF, Asimakopoulos AD, et al. The ETS Homologous Factor (EHF) Represents a Useful Immunohistochemical Marker for Predicting Prostate Cancer Metastasis. Diagnostics. 2022;12:800. 35453848 10.3390/diagnostics12040800 PMC9025154

[17] Richardson G, Kidman S, Langton M, Hermansson A-M. Differences in amylose aggregation and starch gel formation with emulsifiers. Carbohydr Polym. 2004;58:7–13.

[18] Kurata H, Ishigami A, Tokunaga I, Nishimura A. An infant autopsy case of bowel obstruction due to internal abdominal hernia. Leg Med. 2019;38:32–5. 10.1016/j.legalmed.2019.03.006 30927624

[19] Mauriello S, Treglia M, Pallocci M, Bonfiglio R, Giacobbi E, Passalacqua P, et al. Antigenicity Preservation Is Related to Tissue Characteristics and the Post-Mortem Interval: Immunohistochemical Study and Literature Review. Healthcare. 2022;10:1495. 36011152 10.3390/healthcare10081495 PMC9408092

[20] Kontopidis G, Holt C, Sawyer L. Invited Review: β-Lactoglobulin: Binding Properties, Structure, and Function. J Dairy Sci. 2004;87:785–96. 15259212 10.3168/jds.S0022-0302(04)73222-1

[21] Mäkinen-Kiljunen S, Sorva R. Bovine β-lactoglobulin levels in hydrolysed protein formulas for infant feeding. Clin Exp Allergy. 1993;23:287–91. 8319125 10.1111/j.1365-2222.1993.tb00324.x

[22] Orenstein SR. An overview of reflux-associated disorders in infants: apnea, laryngospasm, and aspiration. Am J Med. 2001;111:60–3. 10.1016/s0002-9343(01)00823-3 11749927

[23] Curien-Chotard M, Jantchou P. Natural history of gastroesophageal reflux in infancy: new data from a prospective cohort. BMC Pediatr. 2020;20:152. 32264869 10.1186/s12887-020-02047-3 PMC7137340

[24] Czinn SJ, Blanchard S. Gastroesophageal Reflux Disease in Neonates and Infants. Pediatr Drugs. 2013;15:19–27. 10.1007/s40272-012-0004-2 23322552

[25] United States Consumer Product Safety Commission. Infant deaths prompt CPSC warning about sling carriers for babies. 2010. https://www.cpsc.gov/Newsroom/News-Releases/2010/Infant-Deaths-Prompt-CPSC-Warning-About-Sling-Carriers-for-Babies. Accessed 27 Aug 2024.

[26] Moon RY. SIDS and Other Sleep-Related Infant Deaths: Expansion of Recommendations for a Safe Infant Sleeping Environment. Pediatrics. 2011;128:e1341–67. 22007004 10.1542/peds.2011-2284

